# Dual-Energy CT muscle fat fraction as a new imaging biomarker of body composition and survival predictor in critically ill patients

**DOI:** 10.1007/s00330-024-10779-4

**Published:** 2024-05-22

**Authors:** Jennifer Erley, Kevin Roedl, Ann-Kathrin Ozga, Geraldine de Heer, Niklas Schubert, Julia Breckow, Christoph Burdelski, Enver Tahir, Stefan Kluge, Tobias B. Huber, Jin Yamamura, Gerhard Adam, Isabel Molwitz

**Affiliations:** 1 Department of Diagnostic and Interventional Radiology and Nuclear Medicine, University Medical Center Hamburg-Eppendorf, Hamburg, Germany; 2 Department of Intensive Care Medicine, University Medical Center Hamburg-Eppendorf, Hamburg, Germany; 3 Institute of Medical Biometry and Epidemiology, University Medical Center Hamburg-Eppendorf, Hamburg, Germany; 4 III. Department of Medicine, University Medical Center Hamburg-Eppendorf, Hamburg, Germany; 5 Hamburg Center for Kidney Health (HCKH), University Medical Center Hamburg-Eppendorf, Hamburg, Germany

**Keywords:** Computed tomography, Spectral-CT, Myosteatosis, Body composition, Critical illness

## Abstract

**Objective:**

To analyze changes in the muscular fat fraction (FF) during immobilization at the intensive care unit (ICU) using dual-energy CT (DECT) and evaluate the predictive value of the DECT FF as a new imaging biomarker for morbidity and survival.

**Methods:**

Immobilized ICU patients (*n* = 81, 43.2% female, 60.3 ± 12.7 years) were included, who received two dual-source DECT scans (CT1, CT2) within a minimum interval of 10 days between 11/2019 and 09/2022. The DECT FF was quantified for the posterior paraspinal muscle by two radiologists using material decomposition. The skeletal muscle index (SMI), muscle radiodensity attenuation (MRA), subcutaneous-/ visceral adipose tissue area (SAT, VAT), and waist circumference (WC) were assessed. Reasons for ICU admission, clinical scoring systems, therapeutic regimes, and in-hospital mortality were noted. Linear mixed models, Cox regression, and intraclass correlation coefficients were employed.

**Results:**

Between CT1 and CT2 (median 21 days), the DECT FF increased (from 20.9% ± 12.0 to 27.0% ± 12.0, *p* = 0.001). The SMI decreased (35.7 cm^2^/m^2^ ± 8.8 to 31.1 cm^2^/m^2^ ± 7.6, *p* < 0.001) as did the MRA (29 HU ±  10 to 26 HU ± 11, *p* = 0.009). WC, SAT, and VAT did not change. In-hospital mortality was 61.5%. In multivariable analyses, only the change in DECT FF was associated with in-hospital mortality (hazard ratio (HR) 9.20 [1.78–47.71], *p* = 0.008), renal replacement therapy (HR 48.67 [9.18–258.09], *p* < 0.001), and tracheotomy at ICU (HR 37.22 [5.66–245.02], *p* < 0.001). Inter-observer reproducibility of DECT FF measurements was excellent (CT1: 0.98 [0.97; 0.99], CT2: 0.99 [0.96–0.99]).

**Conclusion:**

The DECT FF appears to be suitable for detecting increasing myosteatosis. It seems to have predictive value as a new imaging biomarker for ICU patients.

**Clinical relevance statement:**

The dual-energy CT muscular fat fraction appears to be a robust imaging biomarker to detect and monitor myosteatosis. It has potential for prognosticating, risk stratifying, and thereby guiding therapeutic nutritional regimes and physiotherapy in critically ill patients.

**Key Points:**

- *The dual-energy CT muscular fat fraction detects increasing myosteatosis caused by immobilization.*
- *Change in dual-energy CT muscular fat fraction was a predictor of  in-hospital morbidity and mortality.*
- *Dual-energy CT muscular fat fraction had a predictive value superior to established CT body composition parameters.*

## Introduction

Critically ill patients in the intensive care unit (ICU) commonly suffer from sarcopenia [1]. Sarcopenia is defined as low muscle strength with loss of muscle mass or loss of muscle quality [2]. It is primarily caused by a high age (primary sarcopenia). Secondary sarcopenia can be caused by risk factors, such as malnutrition, inflammation, cardiopulmonary complications, and inactivity, all of which are frequent in critically ill patients [3]. Sarcopenia results in adverse health outcomes, such as falls, fractures, metabolic syndrome, depression, postoperative complications, and poor overall survival [4]. If detected at an early stage, irreversible muscle wasting can be prevented by regular physical activity, neuromuscular electrical stimulation in immobilized patients, and nutritional support [5]. The effective treatment of sarcopenia leads to a reduced length of ICU- and hospital stay, as well as decreased rates of delirium and sedation in the critically ill [6, 7].

Low muscle strength is indicative of sarcopenia [1]. The diagnosis should then be confirmed by measuring reduced muscle mass or muscle quality [1]. Muscle strength is usually clinically assessed, e.g., by measuring the hand grip strength [1]. However, this requires awake and cooperative patients and is thus challenging in the ICU [7]. Similarly, the quantification of both muscle quantity and quality is frequently hindered in critically ill patients. Muscle mass can be quantified using bioelectrical impedance analysis (BIA), ultrasonography, computed tomography (CT), or magnetic resonance imaging (MRI). However, BIA and ultrasonography are influenced by muscular edema, operator-dependent, and of disputed diagnostic accuracy [8]. Even if not the most common in clinical routine, MRI and CT are thus considered the gold standard [9]. In ICU patients, CT is the most convenient imaging modality, as these patients often require repetitive CT examinations for clinical reasons. Also, the use of MRI is frequently limited due to in-part ferromagnetic medical equipment in ICU patients. With CT, the skeletal muscle mass can be assessed based on the appendicular, psoas, or whole abdominal muscle area [2].

Muscle quality is defined as micro- and macroscopic aspects of muscle architecture and composition [1], which explain muscle strength per unit size of muscle mass. It is dependent on the deposition of fat inside the skeletal muscle [10], the so-called myosteatosis [10]. Myosteatosis is closely linked to sarcopenia [11], despite being recognized as a distinctive disease [12]. Myosteatosis is caused by various mechanisms, such as the adipogenic conversion of precursor stem cells due to increased glucocorticoid levels [12, 13]. The prognostic relevance of myosteatosis has been shown in various patient groups, such as in different cancer entities [14]. Myosteatosis commonly serves as a parameter of muscle quality for body composition analyses in CT and MRI [15]. To assess myosteatosis with MRI, it is necessary to prospectively select suited sequences for fat quantification. In CT, only indirect and easily biased options existed to determine myosteatosis. Besides subjective grading of muscle morphology [16], most commonly, the CT density, the so-called muscle radiodensity attenuation (MRA), is applied to assess myosteatosis and, thus, muscle quality. Even if the MRA has a predictive value for many entities [12, 17], its use is limited due to the impact of contrast agent on muscle density.

Recently, it has thus been proposed to use dual-energy CT (DECT) material quantification to directly quantify the muscular fat content (FF) unbiased by contrast agent [18]. Good agreement between the DECT FF and MRI chemical shift relaxometry was demonstrated [18], as well as the ability to distinguish between different MR grades of fat infiltration [19]. However, the applicability of the DECT FF to monitor myosteatosis in a clinical cohort and its potential predictive value were not investigated, yet.

Hence, this study aimed to investigate changes in the muscle status in immobilized ICU patients using DECT FF and conventional CT parameters of muscle quantity (skeletal muscle index (SMI)) and quality (MRA). Secondly, we evaluated the predictive value of conventional CT parameters and the DECT FF as a new imaging biomarker for morbidity and survival.

## Methods

This retrospective observational study was approved by the local ethics committee (Ärztekammer Hamburg, PV7006-4406-BO-ff). All analyses were conducted in accordance with the Declaration of Helsinki and in compliance with local ethical guidelines. The article is reported in accordance with the STROBE guidelines [20].

### Study population

Included were ICU patients who a) were intubated and thus immobilized, and b) received two contrast-enhanced abdominal DECT scans within a minimum time interval of ten days between November 2019 and September 2022. The minimum time interval of ten days was chosen, as this study aimed to investigate the suitability of the DECT FF to detect changes in muscle status in comparison to other CT body composition parameters. Thus, a sufficiently long period of immobilization was necessary for changes in muscle status to occur. According to the literature, muscle changes can occur very quickly, but have likely occurred within 14 days (with variations depending on the collective and the muscle group) [21]. Exclusion criteria were a) age under 18 years, b) discharge from the ICU between CT scans, and c) artifacts from metal implants that reduced CT image quality. Other parameters that may impact image quality, such as ascites or anasarca were noted, but patients were not excluded, as the DECT FF was nevertheless expected to be applicable.

### DECT image acquisition

All patients received DECT scans using a dual-source CT scanner (SOMATOM Force, Siemens Healthineers, Erlangen, Germany). Scan parameters were 100 kV and 150 kV with a tin filter, pitch 0.5, collimation 0.6 mm, slice thickness 1 mm (reconstructed slice thickness: 5 mm), pixel size 0.6 × 1 mm. Images were acquired 80 seconds after injection of 80 mL Iomeprol contrast agent (Imeron 350 M, Bracco IMAGING, Milan, Italy).

### DECT muscle fat quantification

The DECT FF was quantified using the standard software of the CT scanner’s manufacturer (syngo.via, Siemens Healthineers, Erlangen, Germany). Details of the postprocessing have been previously described in detail and validated by Molwitz et al [18]. To summarize, virtual noncontrast images were created, based on three material decomposition for soft tissue, iodine, and fat using the syngo.via *“Liver VNC”* tool. With the “*Liver fat map”* tool, quantified fat values and a color-coded fat concentration map can be displayed as an overlay on the CT grey-scale images. Regions of interest (ROI) (median 8.1 cm^2^) were drawn on transverse CT images with fat map overlays by contouring the inner circumference of the posterior paraspinal muscle on both sides of the spine at the height of the third lumbar vertebra (L_3_). The height of L_3_ was chosen as the muscle area at this height has been demonstrated to correlate best with the whole-body muscle mass [22]. It is thus the standard measurement height for body composition analyses and allows direct comparison to other studies. ROIs were defined for each patient on three adjacent slices to enhance the robustness of the measurement results. The fat fraction (%) was noted for each ROI and averaged per side and per patient. All DECT analyses were performed independently by two radiologists (4 and 2 years of experience).

### Assessment of conventional CT body composition parameters

**Fig. 1:**
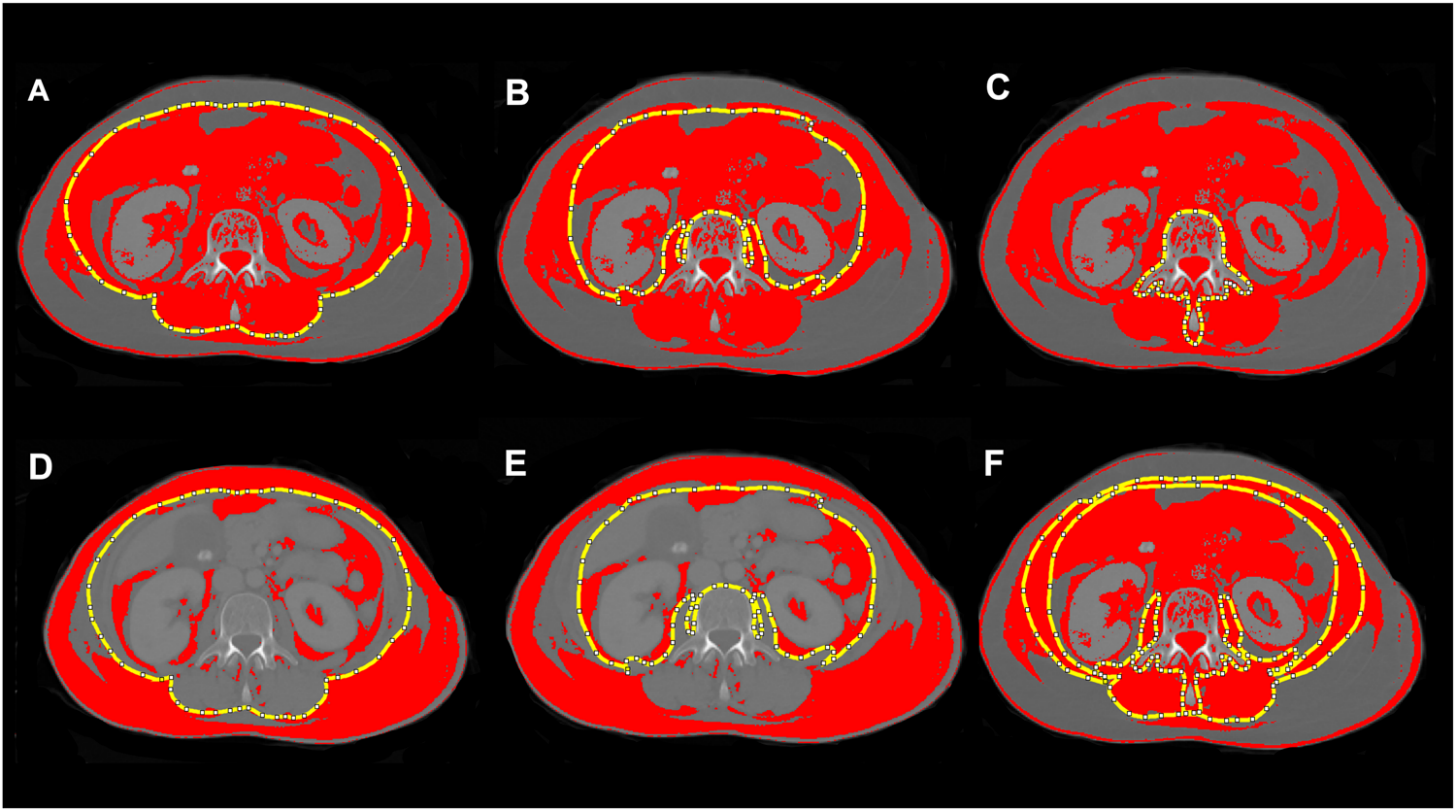
Postprocessing contours to quantify conventional CT body composition parameters. Displayed is the outer (**A**) and inner (**B**) perimeter of the abdominal and paraspinal muscle, and of the third lumbar vertebra (**C**), as well as the whole abdominal muscle area (**F**) each with the muscle-specific threshold of –29 to +150 HU. With a fat-specific threshold of −150 to −30 HU, (**D**) displays the outer and (**E**) the inner muscle perimeter. The skeletal muscle index is then given according to Gomez-Perez et al [23] by subtracting **B** and **C** from **A**. The muscle radiodensity attenuation is given by **F**. The subcutaneous adipose tissue is given by the area outside the defined muscle perimeter in **D**, and the visceral fat by the area within the perimeter in **E**

For each patient, a transverse CT image at the mid-height of L_3_ was exported from the radiological Picture Archiving and Communication system (PACS, GE Centricity, Milwaukee, USA). For further processing, the open-source software Image J (National Institutes of Health) was used in line with a step-by-step guide previously published by Gomez-Perez et al [23]. Figure 1 shows these post-processing steps in an exemplary study patient. First, the circumference of the inner and outer abdominal and paraspinal musculature, as well as the circumference of L_3_ was outlined. After application of a muscle-specific threshold (−29 to +150 Hounsfield units [HU]), the area within the inner perimeter of the musculature (Fig. 1B) and of the vertebra (Fig. 1C) were subtracted from the area within the outer perimeter of the musculature (Fig. 1A). The derived skeletal muscle area was divided by square body height (m) to provide the SMI (cm^2^/m^2^). The MRA (HU) was given by the mean density of the whole abdominal muscle area (Fig. 1F). The waist circumference (WC, cm) was assessed by contouring the body circumference at the height of L_3_. The subcutaneous adipose tissue area (SAT, cm^2^) was calculated by subtracting the area within the outer muscle circumference (Fig. 1D) from the whole-body area after application of a fat-specific threshold (−150 to −30 HU). The visceral adipose tissue area (VAT, cm^2^) was derived from the area within the inner muscle perimeter (Fig. 1E) using the same fat-specific threshold.

### Assessment of clinical parameters

Dates of hospital and ICU admission/dismissal, or death were noted. Reasons for ICU admission were determined and categorized in post-surgery vs. other medical indications. The “Simplified Acute Physiology Score II” (SAPS II), “Sequential Organ Failure Assessment” (SOFA), and the “Charlson Comorbidity Index” (CCI) were assessed at ICU admission. Patient diseases that were assessed as part of the CCI were clustered into the following categories: known malignancies (solid tumor, leukemia, or lymphoma), chronic diseases (chronic obstructive pulmonary disease; chronic heart failure defined as exertional or paroxysmal nocturnal dyspnea which responded to digitalis, diuretics, or afterload-reducing agents; liver failure which is categorized into mild (no portal hypertension), moderate (with portal hypertension), and severe (with portal hypertension and variceal bleeding history); renal disease defined as moderate (creatinine > 3 mg/dL) or severe (on dialysis or status post kidney transplant)). Laboratory measurements (platelets, lactate, bilirubin, creatinine) were noted at the time of ICU admission. Also, data were collected on therapeutic measurements (invasive ventilation, tracheotomy, renal replacement therapy, use of vasopressors, and parenteral nutrition).

### Statistical analysis

Continuous data are represented using mean with standard deviation if normally distributed or median with range (min-max) and categorical data via absolute and relative frequencies. The inter-observer variability was determined using intraclass correlation coefficients (two-way mixed, absolute agreement) (ICC). Due to the different units of measurement, the change in DECT FF, SMI, and MRA from CT1 to CT2 was standardized using a z-transformation. Further, the measurement difference was then divided by the individual time difference between CT1 and CT2 for each patient. The values of the MRA and SMI (where a decrease was expected) were multiplied with –1 to make them comparable to the DECT FF measurements. Spearman correlation coefficients (continuous variables) or point biserial correlation (categorical with continuous variables) were assessed. Linear mixed models were employed, including the CT parameters at CT1, the change in CT parameters standardized to the varying time intervals between CT1 and CT2, and the following independent variables: age, sex, body mass index (BMI), the reason for ICU stay (surgical vs. non-surgical), and diseases (malignancies, chronic diseases, chronic inflammatory diseases, renal diseases). The random effect was the patient. Uni- and multivariable Cox-regression analyses were used to test the association between the changes in body composition parameters standardized to the varying time intervals between CT1 and CT2 with in-hospital mortality, new renal replacement therapy (including sex, age, BMI, SAPS II, SOFA, CCI, creatinine at admission, and known renal disease) and tracheotomy at ICU (including sex, age, BMI, SAPS II, SOFA, and CCI). The clinical scoring systems (SAPS II, SOFA, and CCI) were included as independent variables instead of outcome variables as infections or sepsis (SAPS II, SOFA), or comorbidities (CCI) were already commonly present at ICU admission. Model assumptions were checked graphically via histograms of residuals for linear models and Schoenfeld residuals, as well as martingale residuals for the Cox model. There were no missing data. No adjustment for multiple testing was conducted due to the explorative design of the study. Because of the explorative design, *p* values are descriptive. Statistical analyses were conducted using R version 4.2.3 and SPSS (Version 28.0.1.1, IBM, Armonk, NY).

## Results

### Study Population

A total of 81 patients were included in the final study population (35 (43.2%) female, average age 60.3 years ± 12.7). The median time difference between CT1 and CT2 was 21 days (range: 10–195). The median time difference from ICU admission to CT1 was 8 days (range: 0–175). At ICU admission (CT 1), ascites or anasarca were present in 56 patients (68%). Patients spent a median time of 53 days (range: 13–321) in the ICU. Of all patients, 48 (61.5%) died during the hospital stay, and 45 died in the ICU. Table 1 illustrates patient characteristics as well as laboratory values at ICU admission.

**Table 1:** Demographic characteristics of the patient population

**Patient characteristics**	
Female, *n* (%)	35 (43.2)
Age (years), mean (sd)	60.3 (12.7)
BMI (kg/m^2^), mean (sd)	25.1 (5.5)
Time spent in hospital (days), median (range)	66 (14–321)
Time spent in ICU (days), median (range)	53 (13–321)
Admission post-surgery, *n* (%)	29 (35.8)
Admission for other clinical reasons, *n* (%)	52 (64.2)
SAPS II at admission (points), mean (sd)	44.9 (11.5)
SOFA at admission (points), mean (sd)	10.0 (4.1)
CCI at admission (points), mean (sd)	3.6 (3.0)
In-hospital death, *n* (%)	48 (61.5)
ICU-death, *n* (%)	45 (56.2)
**Comorbidities**	
Myocardial Infarction, *n* (%)	23 (28.4)
Heart failure, *n* (%)	14 (17.3)
Peripheral artery occlusive disease, *n* (%)	9 (11.2)
Cerebrovascular artery occlusive disease, *n* (%)	9 (11.1)
Chronic pulmonary disease, *n* (%)	19 (23.5)
Connective tissue disease, *n* (%)	12 (14.8)
Ulcer, *n* (%)	9 (11.1)
Liver disease, *n* (%)	12 (14.8)
Diabetes mellitus, *n* (%)	6 (7.4)
Hemiplegia, *n* (%)	2 (2.5)
Renal disease, *n* (%)	20 (24.7)
Malignancies, *n* (%)	28 (34.6)
Chronic diseases, *n* (%)	48 (59.3)
Chronic-inflammatory diseases, *n* (%)	12 (14.8)
**ICU treatment regimes**	
Renal replacement therapy, *n* (%)	64 (79.0)
Invasive ventilation, *n* (%)	81 (100)
Length of invasive ventilation (days), median (range)	34 (0–210)
Tracheotomy, *n* (%)	57 (70.4)
Weaning, *n* (%)	26 (32.5)
Vasopressors, *n* (%)	80 (98.8)
Parenteral nutrition, *n* (%)	79 (97.5)
Enteral nutrition, n (%)	79 (97.5)
**Laboratory values at ICU admission**	
Lactate (mg/dL), mean (sd)	2.4 (2.4)
Thrombocytes (10^3^/μL), mean (sd)	196.4 (161.4)
Bilirubin (mg/dL), mean (sd)	2.3 (4.4)
Creatinine (mg/dL), mean (sd)	1.9 (1.4)

*sd* standard deviation

### Body composition results of CT1

On average, male patients showed a −6.7% lower DECT FF than female patients [95% confidence interval (CI) −12.2; −1.2], *p* = 0.018). The SMI was higher in male patients (4.5 cm^2^/m^2^ [0.7; 8.2], *p* = 0.020), as was the VAT (68.4 cm^2^/m^2^ [34.4; 102.4], *p* < 0.001). A higher age was associated with a higher DECT FF (0.3% [0.1; 0.5], *p* = 0.014) and VAT (1.6 cm^2^ [0.3; 2.9], *p* = 0.021). A higher BMI was associated with increased WC, SAT, and VAT (all *p* < 0.001). SMI, MRA, and DECT FF were not influenced by BMI. SMI at CT1 was lower if patients received renal replacement therapy (−8.2 cm^2^/m^2^ [−13.7; −2.7], *p* = 0.004). Table 2 shows the effects of different patient characteristics on measurement values of the DECT FF, MRA, and SMI at CT1, Supplementary Table 1 shows the effect of these variables on the WC, SAT, and VAT.

**Table 2:** Results of the linear mixed models, showing the association between patient characteristics and the DECT FF, MRA, and SMI at CT1

	DECT FF (%)		MRA (HU)		SMI (cm^2^/m^2^)	
	B [95%CI]	*p*	B [95%CI]	*p*	B [95%CI]	*p*
Male sex	−**6.70** **[−12.2;** −**1.19]**	**0.018**	2.51 [−2.12; 7.14]	0.283	**4.48** **[0.73;** **8.24]**	**0.020**
Age	**0.27** **[0.06; 0.49]**	**0.014**	−**0.23** **[−0.41;** −**0.05]**	**0.012**	0.13 [−0.02; 0.27]	0.093
BMI	0.22 [−0.27; 0.71]	0.370	−0.32 [−0.73; 0.10]	0.132	0.29 [−0.05; 0.62]	0.091
Admission post-surgery	1.09 [−4.49; 6.67]	0.698	−1.59 [−6.28; 3.11]	0.503	3.69 [−0.12; 7.50]	0.057
Malignancies	0.64 [−5.44; 6.72]	0.835	−1.10 [−6.21; 4.01]	0.669	−2.83 [V6.97; 1.32]	0.178
Chronic diseases	1.23 [−5.07; 7.52]	0.699	−3.28 [−8.57; 2.02]	0.221	−1.38 [−5.67; 2.92]	0.524
Chronic inflammatory diseases	−2.20 [−10.20; 5.79]	0.584	2.66 [−4.06; 9.38]	0.433	−1.52 [−6.98; 3.93]	0.579
Renal diseases	−2.36 [−9.57; 4.86]	0.517	0.37 [−5.69; 6.44]	0.903	0.16 [−4.76; 5.08]	0.948
Renal replacement therapy	2.32 [−5.79; 10.42]	0.571	0.60 [−6.21; 7.41]	0.861	−**8.20** **[−13.73;** −**2.67]**	**0.004**

Males showed a lower DECT FF and a higher SMI than females. A higher age was associated with a higher DECT FF, and MRA. The SMI, MRA, and DECT FF were not associated with the BMI

Results of statistic relevance, defined as a *p* value below 0.05, are highlighted in bold

*B* regression coefficient

### Change in body composition between CT1 and CT2

**Fig. 2:**
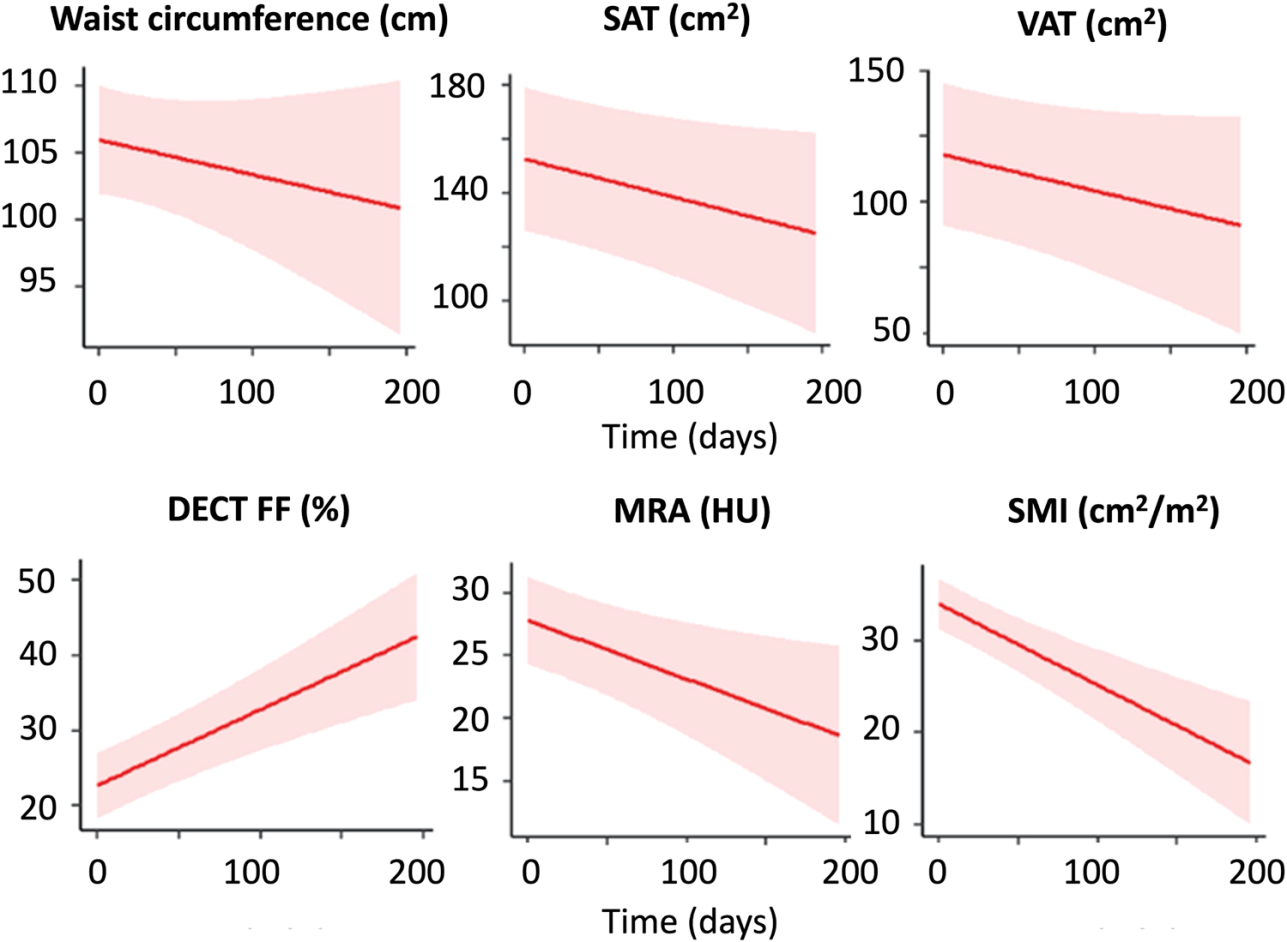
Change of CT parameters during immobilization at the intensive care unit. The skeletal muscle index (SMI) as a measure of muscle mass decreased over time. The DECT FF increased and the muscle radiodensity attenuation (MRA) in HU decreased, both demonstrating progressive myosteatosis. Decreases of waist circumference (WC), subcutaneous and visceral adipose tissue area (SAT and VAT) were less pronounced with large standard deviations

**Fig. 3:**
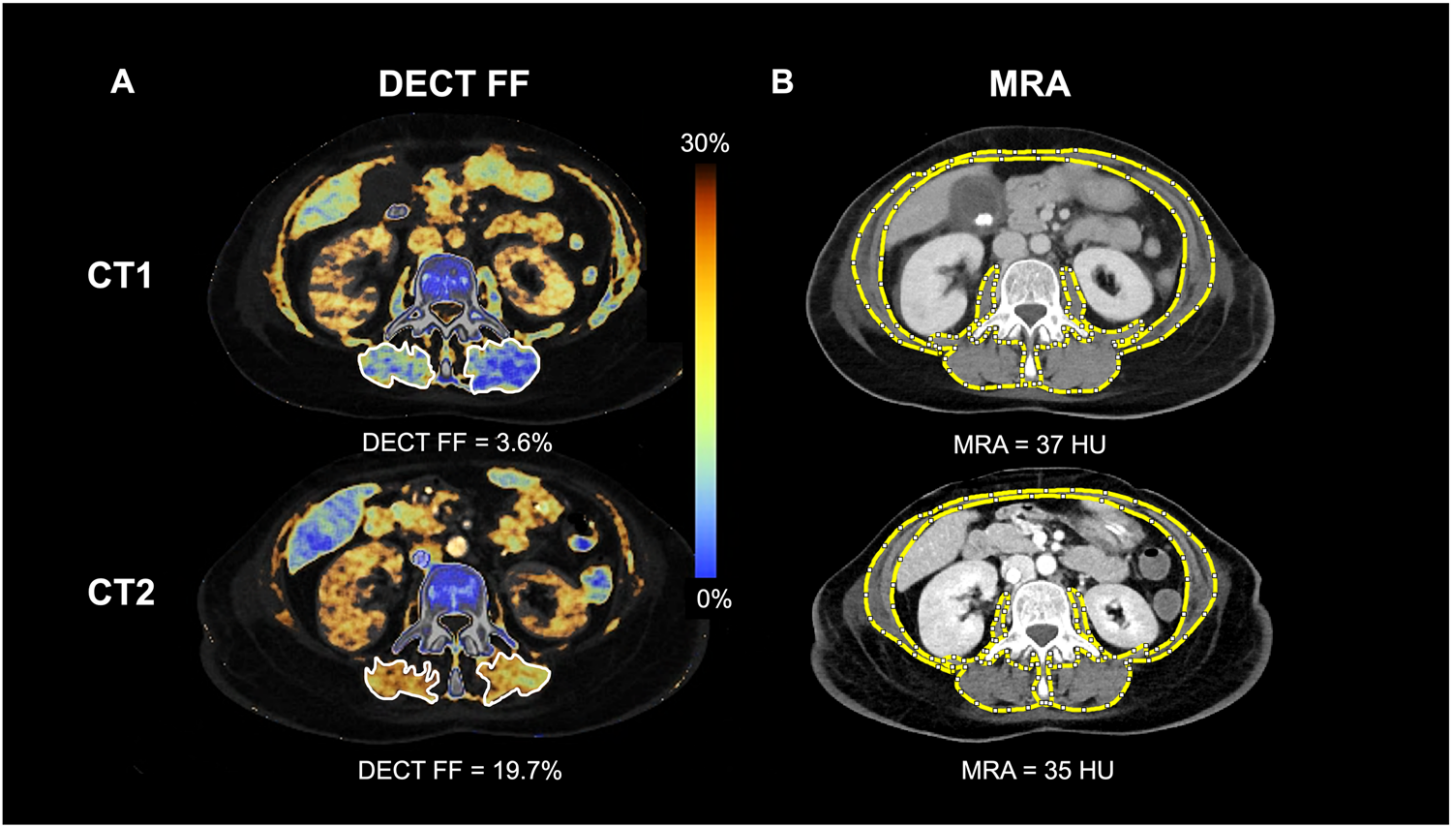
Changes of DECT FF (**A**) and MRA (**B**) during immobilization of a 56-year-old female patient with pneumonia. This patient received mechanical ventilation, parenteral nutrition, and renal replacement therapy at intensive care unit, and died 3 days after CT2. The time interval between CT1 and CT2 was 22 days. A marked increase in the DECT FF and thus an increase of myosteatosis was observed. The change of the muscle radiodensity attenuation (MRA) was less pronounced, potentially due to the impact of the contrast agent on the MRA. The skeletal muscle index (not depicted) also decreased from 34.5 to 28.1 cm^2^/m^2^

The effect of independent variables (e.g., sex, comorbidities) on the change of CT body composition parameters standardized to the varying time intervals between CT1 and CT2 is shown in Table 3 and Supplement, Table 2. The changes in CT parameters over time are schematically illustrated in Fig. 2. The DECT FF increased from 20.9% ± 12.0 to 27.0% ± 12.0 (effect size: 0.08 [0.03; 0.12], *p* = 0.001), while the MRA decreased from 29 HU ± 10 to 26 HU ± 11 with a proportionally smaller effect size (effect size: 0.05 [−0.08; −0.01], *p* = 0.009). The SMI also decreased from 35.7 ± 8.8 to 31.1 ± 7.6 cm^2^/m^2^ (effect size: −0.09 [−0.12; −0.05], *p* < 0.001). WC, SAT, and VAT did not change significantly. Figure 3 portrays the DECT FF and MRA at CT1 and CT2 in an exemplary 56-year-old female patient with severe pneumonia. The time interval between CT1 and CT2 in this patient was 22 days. The DECT FF increased from 3.6% at CT1 to 19.7% at CT2, while the MRA decreased from 37 to 35 HU.

**Table 3:** Results of the mixed linear model investigating the effect of sex, age, BMI, or comorbidities on the change of the DECT FF, SMI, and MRA standardized to the varying time intervals between CT1 and CT2

	DECT FF (%)		MRA (HU)		SMI (cm^2^/m^2^)	
	Effect size [95%CI]	*p*	Effect size [95%CI]	*p*	Effect size [95%CI]	*p*
Time (from CT1 to CT2)	**0.08** **[0.03; 0.12]**	**0.001**	−**0.05** **[−0.08;** −**0.01]**	**0.009**	−**0.09** **[−0.12;** −**0.05]**	**< 0.001**
Male sex	−**7.25** **[−12.43;** −**2.08]**	**0.006**	2.82 [−1.44; 7.08]	0.193	3.32 [−0.03; 6.68]	0.052
Age	**0.27** **[0.06;** **0.47]**	**0.010**	−**0.26** **[−0.42;** −**0.09]**	**0.003**	0.11 [−0.02; 0.24]	0.110
BMI	0.42 [−0.06; 0.89]	0.084	−**0.55** **[−0.94;** −**0.16]**	**0.006**	0.12 [−0.18; 0.43]	0.429
Admission post-surgery	0.65 [−4.77; 6.08]	0.812	−2.79 [−7.26; −1.67]	0.218	**4.39** **[0.87; 7.90]**	**0.015**
Malignancies	−0.06 [−5.85; 5.73]	0.983	−1.50 [−6.15; 3.15]	0.526	−1.74 [−5.40; −1.92]	0.350
Chronic diseases	1.48 [−4.45; 7.42]	0.623	−3.88 [−8.77; −1.00]	0.119	−0.24 [−4.08; 3.61]	0.903
Chronic inflammatory diseases	0.13 [−7.28; 7.55]	0.972	0.78 [−5.33; 6.89]	0.801	−2.16 [−6.97; 2.65]	0.376
Renal diseases	−2.43 [−9.19; 4.32]	0.478	0.99 [−4.58; 6.55]	0.727	−0.94 [−5.32; 3.44]	0.673

The DECT FF increased and MRA decreased between CT1 and CT2 as indicators of progressive myosteatosis. The increase in DECT FF was proportionally greater. The SMI, as a parameter of muscle mass, decreased over time

Results of statistic relevance, defined as a *p* value below 0.05, are highlighted in bold

### Correlation between CT body composition parameters

The DECT FF showed a good correlation with the MRA (*r* = −0.75 at CT1; *r* = −0.72 at CT2) and almost no correlation with the SMI (*r* = −0.18 at CT1; *r* = −0.21 at CT2). The increase in DECT FF also correlated well with the decrease in MRA (*r* = 0.61), while the correlation with the decrease of the SMI was weak (*r* = 0.23).

### Association between CT parameters and morbidity/mortality

**Fig. 4:**
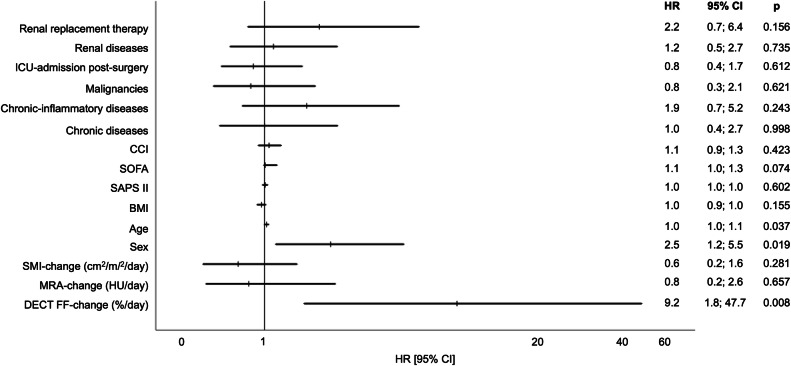
Association between the clinical and CT parameters with in-hospital mortality. The change of the DECT FF per day was associated with in-hospital mortality, while neither the changes of the skeletal muscle index (SMI), of the muscle radiodensity attenuation (MRA) nor the clinical parameters and scores were

The relationships between the changes of the DECT FF, MRA, and SMI per day, standardized to the varying time intervals between CT1 and CT2, with in-hospital mortality after CT2 are shown in Table 4 and Fig. 4. In the multivariable analyses, the increase of the DECT FF per day was associated with in-hospital mortality (hazard ratio (HR): 9.20 [1.78; 47.71], *p* = 0.008), while the changes of the MRA and SMI were not. Of all other investigated parameters, only a higher age (HR: 1.03 [1.00; 1.07], *p* = 0.037) and male sex (HR: 2.52 [1.17; 5.45], *p* = 0.019) were associated with mortality (Fig. 4). In the univariable analysis, both, changes of the DECT FF and the MRA were significantly associated with mortality, but the increase in DECT FF showed the highest HR. The absolute measurement values of the DECT FF, MRA, or SMI at CT1 or CT2 were not associated with in-hospital mortality.

**Table 4:** Results of the Cox regression analysis on the association between the changes of the DECT FF, MRA, and SMI (standardized to the varying time intervals between CT1 and CT 2) and in-hospital-mortality after CT2

	Univariable		Model 1		Model 2		Model 3	
**In-Hospital Mortality**	**HR** **[95% CI]**	***p***	**HR** **[95% CI]**	***p***	**HR** **[95% CI]**	***p***	**HR** **[95% CI]**	***p***
**DECT FF change (%/day)**	**3.15** **[1.32; 7.50]**	**0.010**	**7.09** **[1.67; 30.00]**	**0.008**	**9.93** **[2.31; 42.7]**	**0.002**	**9.20** **[1.78; 47.71]**	**0.008**
**MRA change (HU/day)**	**2.30** **[1.10; 4.81]**	**0.027**	1.08 [0.33; 3.56]	0.899	0.93 [0.29; 3.05]	0.909	0.75 [0.22; 2.63]	0.657
**SMI change (cm**^**2**^**/m**^**2**^**/day)**	0.84 [0.35; 1.99]	0.689	0.56 [0.23; 1.41]	0.220	0.61 [0.24; 1.59]	0.312	0.55 [0.19; 1.63]	0.281

The relative increase in DECT FF was associated with in-hospital mortality, while the changes of the SMI and MRA were not

Results of statistic relevance, defined as a *p* value below 0.05, are highlighted in bold

Model 1 = Multivariable model including DECT FF, MRA, SMI, sex, age, and BMI

Model 2 = Multivariable model including DECT FF, MRA, SMI, sex, age, BMI, and SOFA-/ SAPS II-/CCI- score at ICU admission

Model 3 = Multivariable model including DECT FF, MRA, SMI, sex, age, BMI, SOFA-/ SAPS II-/CCI- score at ICU admission, operative reason for ICU-admission, malignancies, chronic diseases, chronic inflammatory diseases, renal diseases, and renal replacement therapy

The relationships between the changes of the DECT FF, MRA, and SMI per day standardized to the varying time intervals between CT1 and CT2 and the incidence of a new renal replacement therapy or tracheotomy at ICU are shown in Table 5. In the univariable analysis, the relative increase of the DECT FF showed the strongest association with new renal replacement therapy and tracheotomy at the ICU. In the multivariable models, only the relative increase of the DECT FF per day was associated with the incidence of renal replacement therapy (HR 48.67 [9.18; 258.09], *p* < 0.001) and tracheotomy (HR 37.22 [5.66; 245.02], *p* < 0.001) (Table 5).

**Table 5:** Results of the Cox regression analysis on the association between the changes of the DECT FF, MRA, and SMI (standardized to the varying time intervals between CT1 and CT 2) and the incidence of renal replacement therapy and tracheotomy at the ICU

	Univariable		Multivariable	
	HR [95%CI]	*p*	HR [95% CI]	*p*
**Model 1: new renal replacement therapy**				
DECT FF change (%/day)	**18.40** **[6.42; 52.78]**	**< 0.001**	**48.67** **[9.18; 258.09]**	**< 0.001**
MRA change (HU/day)	**5.12** **[1.99; 13.20]**	**< 0.001**	0.63 [0.18; 2.17]	0.462
SMI change (cm^2^/m^2^/day)	3.12 [1.25; 7.82]	0.015	1.22 [0.40; 3.70]	0.722
**Model 2: new tracheotomy**				
DECT FF change (%/day)	**16.27** **[5.13; 51.58]**	**< 0.001**	**37.22** **[5.66; 245.02]**	**< 0.001**
MRA change (HU/day)	**7.60** **[2.70; 21.40]**	**< 0.001**	1.06 [0.26; 4.28]	0.940
SMI change (cm^2^/m^2^/day)	1.14 [0.42; 3.12]	0.793	0.46 [0.17; 1.23]	0.120

The standardized change in DECT FF showed the strongest association with new renal replacement therapy and tracheotomy at the ICU

Results of statistic relevance, defined as a *p* value below 0.05, are highlighted in bold

Model 1 = Multivariable analysis including DECT FF, MRA, SMI, sex, age, BMI, SOFA-/SAPS II-/ CCI-score at ICU admission, known kidney diseases, and creatinine at admission

Model 2 = Multivariable analysis including DECT FF, MRA, SMI, sex, age, BMI, and SOFA-/ SAPS II-/ CCI-score at ICU admission

### Inter-observer reproducibility

The inter-observer reproducibility of DECT FF measurements was almost perfect (ICC: 0.98 [0.97; 0.99] at CT1 and 0.99 [0.96; 0.99] at CT2).

## Discussion

While the DECT FF had been used for fat quantification in the liver, bone marrow, and adrenal glands [24], it has only recently been proposed as a new biomarker of myosteatosis [18, 25, 26]. This retrospective observational study was the first to investigate changes in the muscle status with DECT FF and to evaluate its predictive value as a new imaging biomarker compared to conventional CT body composition parameters. The major findings of this study were that a) myosteatosis, measured by the DECT FF and MRA, increased during immobilization in the ICU while the SMI and thus muscle mass decreased and b) the increase of the DECT FF was proportionally greater than the decrease of the MRA. Also, c) only the standardized increase in DECT FF was associated with the incidence of new renal replacement therapy and tracheotomy at ICU, as well as with in-hospital mortality, while conventional CT parameters were not.

During immobilization at the ICU, a degradation of muscle quantity and quality (as indicated by muscular fat content [27]) was expected. While the decrease in muscle mass (SMI) is often investigated in critical illness [28, 29], myosteatosis is a poorly explored imaging aspect of muscle weakness in critically ill patients. In this study, the muscle mass (SMI) and muscle density (MRA) decreased from CT1 to CT2, while the DECT FF increased. Previous studies similarly reported a decrease in SMI and MRA over time [30, 31]. However, this was the first time that the DECT FF was applied as a biomarker of myosteatosis in critically ill patients. Interestingly, the changes of the DECT FF and MRA were independent of the BMI. Although myosteatosis is often described in the context of obesity [32], it has been found to develop independent of body weight before [33]. In previous studies, fatty muscle infiltration was also independent of muscle mass [11, 34] and preceded muscle atrophy [32]. That would explain the low correlation between DECT FF and SMI in this study.

Although the decrease of the MRA and increase of the DECT FF both indicated progressive myosteatosis, the increase in DECT FF was more pronounced. This is likely, because opposed to the DECT FF, the MRA is influenced by iodinated contrast agent and the contrast timing of the CT scan [35]. Thus, analyses of the MRA are less reliable. Moreover, in many studies, information on the use of contrast agent or scan phase is missing, which hinders study comparability regarding the MRA, as found by a review of Poltronieri et al of 117 studies [15]. In this context, DECT material decomposition is beneficial, because it inherently distinguishes between materials with different atomic numbers, such as iodine and fat [36]. Hence, the quantity of contrast agent in the respective tissue does not bias fat quantification results.

Regarding the clinical patient outcome, in multivariable analyses only the change in DECT FF per day standardized to the varying time interval between CT1 and CT2 was associated with new renal replacement therapy and tracheotomy at the ICU, as well as in-hospital mortality. These results are contradictory to the previous literature, which reported a relationship between the MRA and mortality in critically ill patients [37, 38]. They may be explained by the inherent limitations of the MRA as discussed above. Still, these results indicate that myosteatosis could have a greater association with morbidity and mortality than muscle quantity. This phenomenon has been described before, e.g., in a meta-analysis including patients with COVID-19 [39], in cancer patients [40, 41], or patients with liver surgery [42, 43]. Likely, intramuscular adipocytes that release adipokines, which lead to insulin resistance, inflammation, and metabolic dysfunction [12, 44], have a negative impact on the overall course of the disease. It is has been described, that myostatosis contributes to insulin abnormalities and diabetes, possibly even independent of obesity [33].

The main limitation of this study is the single center setting with thus limited size and heterogeneity of the patient cohort. Additional investigations with larger patient cohorts are necessary to validate the findings on the potential predictive value of the DECT FF compared also to that of the conventional CT parameters. One would expect that in a larger cohort, not only the change of the DECT FF but also absolute DECT FF values could be defined that predict survival. Theoretically, the predictive value of the DECT FF should at least equal that of the MRA, which has been documented in numerous studies before [17, 45], despite MRA measurements being only semiquantitative and influenced by contrast agent. Concerning the varying time difference between the baseline (CT1) and follow-up CT (CT2), changes of CT parameters were standardized for the respective time interval. Opposed to the SMI and MRA, which were measured based on the whole abdominal muscle as recommended [23] that was not sufficiently feasible for the DECT FF with the syngo.via software. As the muscle status is known to differ between muscle groups [46], in the future, automated segmentation tools should be developed that are compatible with software that allow spectral CT analyses.

## Conclusion

In conclusion, the DECT FF, as a new parameter of myosteatosis, appears to be suited to detect increasing muscular fat content in immobilized critically ill patients. Its change was associated with renal failure, the necessity of tracheotomy, and survival. In contrast-enhanced DECT scans, the DECT FF could be a more robust imaging biomarker of muscular fat contents than the MRA. It has the potential for predictive models on morbidity and mortality, further body composition studies, and clinical use, e.g., for risk stratification and informing treatment decisions on nutritional regimes and physiotherapy.

## Supplementary information

Electronic Supplementary Material

## Abbreviation

95% CI: 95% confidence interval
BIA: Bioelectrical impedance analysis
BMI: Body mass index
CCI: Charlson Comorbidity Index
CT: Computed tomography
DE: Dual-energy
FF: Fat fraction
HR: Hazard ratio
ICU: Intensive care unit
MRA: Muscle radiodensity attenuation (HU)
MRI: Magnetic resonance imaging
SAPS II: Simplified Acute Physiology Score II
SAT: Subcutaneous adipose tissue area (cm^2^)
SMI: Skeletal muscle index (cm^2^/m^2^)
SOFA: Sequential Organ Failure Assessment
VAT: Visceral adipose tissue area (cm^2^)
WC: Waist circumference (cm)
