# Supplementary material for: Dual-Energy CT muscle fat fraction as a new imaging biomarker of body composition and survival predictor in critically ill patients
Source: Eur Radiol. 2024 May 22;34(11):7408–18. doi: 10.1007/s00330-024-10779-4 (PMC11519288; doi:10.1007/s00330-024-10779-4)
Supplement: Supplementary file 1 — Electronic Supplementary Material [file 330_2024_10779_MOESM1_ESM.pdf]

Dual-Energy CT muscle fat fraction as a new imaging biomarker of body composition and survival predictor in critically ill patients

ELECTRONIC SUPPLEMENTARY MATERIAL

Supplement, Table 1 Results of the linear mixed models, showing the association between patient characteristics and the waist circumference (WC), subcutaneous adipose tissue (SAT), and visceral adipose tissue (VAT) at CT1

Male sex was associated with VAT, as was age. BMI was associated with WC, SAT, and VAT.

	**WC (cm)**		**SAT (cm^2)**		**VAT (cm^2)**	
	B [95% CI]	*p*	B [95% CI]	*p*	B [95% CI]	*p*
Male sex	4.69 [-0.82; 10.20]	0.094	-31.44 [-65.37; 2.48]	0.069	**68.42 [34.41; 102.44]**	**<0.001**
Age	0.13 [-0.08; 0.35]	0.221	-0.40 [-1.71; 0.92]	0.548	**1.57 [0.25; 2.89]**	**0.021**
BMI	**1.64 [1.15; 2.13]**	**<0.001**	**16.03 [13.02; 19.04]**	**<0.001**	**10.08 [7.07; 13.10]**	**<0.001**
Admission post-surgery	2.04 [-3.54; 7.62]	0.469	-4.73 [-39.22; 29.75]	0.785	-6.47 [-41.04; 28.11]	0.710
Malignancies	1.35 [-4.72; 7.43]	0.658	-0.00 [-37.33; 37.32]	1.000	16.59 [-20.83; 54.01]	0.380
Chronic diseases	**6.37 [0.08; 12.67]**	**0.047**	-10.51 [-49.05; 28.03]	0.588	27.64 [-10.99; 66.28]	0.158
Chronic inflammatory diseases	0.48 [-7.51; 8.48]	0.905	-18.69 [-67.51; 30.13]	0.448	11.12 [-37.83; 60.06]	0.652
Renal diseases	-0.86 [-8.07; 6.36]	0.814	-4.77 [-48.84; 39.30]	0.830	-16.62 [-60.81; 27.56]	0.456
Renal replacement therapy	-3.32 [-11.43; 4.79]	0.417	-23.21 [-72.70; 26.27]	0.353	-11.99 [-61.60; 37.63]	0.631

Abbreviations: BMI = body mass index, B = regression coefficient, 95% CI = 95% confidence interval.

Supplement, Table 2 Results of the mixed linear model, investigating the effect of gender, age, BMI, or comorbidities on the change of waist circumference (WC), subcutaneous adipose tissue (SAT), and visceral adipose tissue (VAT) standardized to the varying time intervals between CT1 and CT 2

Male sex was associated with a decrease in SAT and an increase in VAT. A higher BMI was associated with an increase in WC, SAT and VAT.

	**WC (cm)**		**SAT (cm^2)**		**VAT (cm^2)**	
	Effect size [95% CI]	*p*	Effect size [95% CI]	*p*	Effect size [95% CI]	*p*
Time	-0.03 [-0.07; 0.02]	0.295	-0.14 [-0.29; 0.01]	0.062	-0.14 [-0.31; 0.04]	0.126
Male sex	3.67 [-1.22; 8.56]	0.140	**-37.67 [-70.68; -4.66]**	**0.026**	**56.36 [22.74; 89.97]**	**0.001**
Age	0.15 [-0.04; 0.34]	0.131	-0.47 [-1.74; 0.80]	0.466	**1.66 [0.36; 2.96]**	**0.012**
BMI	**1.44 [1.00; 1.89]**	**<0.001**	**15.27 [12.30; 18.25]**	**<0.001**	**9.69 [6.65; 12.72]**	**<0.001**
Admission post-surgery	2.70 [-2.42; 7.82]	0.300	-3.43 [-38.29; 31.43]	0.846	1.95 [-33.56; 37.45]	0.914
Malignancies	1.32 [-4.02; 6.66]	0.625	8.24 [-27.54; 44.02]	0.650	16.99 [-19.45; 53.43]	0.358
Chronic diseases	**6.87 [1.26; 12.47]**	**0.017**	-8.89 [-46.28; 28.51]	0.639	21.91 [-16.17; 60.00]	0.257
Chronic inflammatory diseases	-1.05 [-8.06; 5.96]	0.768	-22.02 [-68.65; 24.62]	0.352	0.64 [-46.86; 48.13]	0.979
Renal diseases	-1.82 [-8.20; 4.57]	0.574	-6.12 [-48.63; 36.40]	0.777	-13.32 [-56.62; 29.97]	0.544

Abbreviations: BMI = body mass index, 95% CI = 95% confidence interval.
